# Brief Interventions via Electronic Health Record Messaging for Population-Based Suicide Prevention: Mixed Methods Pilot Study

**DOI:** 10.2196/21127

**Published:** 2021-04-12

**Authors:** Ursula Whiteside, Julie Richards, Gregory E Simon

**Affiliations:** 1 NowMattersNow.org Bellevue, WA United States; 2 Department of Psychiatry and Behavioral Sciences University of Washington Seattle, WA United States; 3 Kaiser Permanente Washington Health Research Institute Seattle, WA United States; 4 Health Services Department University of Washington Seattle, WA United States

**Keywords:** suicide, suicide prevention, dialectical behavior therapy, caring message, web-based, NowMattersNow.org, prevention

## Abstract

**Background:**

New opportunities to create and evaluate population-based selective prevention programs for suicidal behavior are emerging in health care settings. Standard depression severity measures recorded in electronic medical records (EMRs) can be used to identify patients at risk for suicide and suicide attempt, and promising interventions for reducing the risk of suicide attempt in at-risk populations can be adapted for web-based delivery in health care.

**Objective:**

This study aims to evaluate a pilot of a psychoeducational program, focused on developing emotion regulation techniques via a web-based dialectical behavior therapy (DBT) skills site, including four DBT skills, and supported by secure message coaching, including elements of caring messages.

**Methods:**

Patients were eligible based on the EMR-documented responses to the Patient Health Questionnaire indicating suicidal thoughts. We measured feasibility via the proportion of invitees who opened program invitations, visited the web-based consent form page, and consented; acceptability via qualitative feedback from participants about the DBT program; and engagement via the proportion of invitees who began DBT skills as well as the number of website visits for DBT skills and the degree of site engagement.

**Results:**

A total of 60 patients were invited to participate. Overall, 93% (56/60) of the patients opened the invitation and 43% (26/60) consented to participate. DBT skills website users visited the home page on an average of 5.3 times (SD 6.0). Procedures resulted in no complaints and some participant feedback emphasizing the usefulness of DBT skills.

**Conclusions:**

This study supports the potential of using responses to patient health questionnaires in EMRs to identify a high-risk population and offer key elements of caring messages and DBT adapted for a low-intensity intervention. A randomized trial evaluating the effectiveness of this program is now underway (ClinicalTrials.gov: NCT02326883).

## Introduction

### Background

Significant momentum supports the work of suicide prevention in health care settings as well as the inclusion of people who have personal experience with suicidal thoughts and behaviors, including systems changes initiatives such as Zero Suicide and new national patient care guidelines such as Recommended Standard Care for People With Suicide Risk: Making Health Care Suicide Safe [1-3]. However, there are significant challenges that hamper major advancements in the field. Although research support for clinical and tertiary treatments exists, these treatments are often intended for those who have already made suicide attempts or have been hospitalized for suicide-related reasons and they likely only have a minimal effect on suicide rates of the overall population [4,5]. This may be because of limited availability and postvention focus. Intensive tertiary interventions for suicidal patients, such as dialectical behavior therapy (DBT) and cognitive behavioral therapy (CBT) for suicide prevention, require significant clinical training for clinicians[6,7]. For patients, they are often costly, time intensive, and inaccessible. Empirically supported interventions target individuals who have already engaged in a suicide attempt [8]. Meanwhile, clinical responses to patients who report suicidal ideation generally focus on acute crises and risk management, such as safety planning, emergency room care, and inpatient psychiatric care. Unfortunately, this sometimes leads to coercive interactions because the onus is on the patient to prove that they are no longer suicidal or *at risk* to leave the health care setting [9].

Selective prevention—otherwise known as secondary or indicated prevention—focused on outreach, support, and skills development would allow for a more collaborative approach. Owing to several conceptual, methodological, logistical, and ethical challenges, selective prevention for suicide remains to be relatively unexplored. Effective selective prevention for suicide requires three things: (1) accurate methods for identifying those at risk before a suicide attempt, (2) effective interventions suitable for large-scale delivery, and (3) acceptable and efficient models for population-based delivery.

The Patient Health Questionnaire (PHQ) [10], a depression questionnaire commonly documented in electronic medical records (EMRs), can be used to identify those greater short-term (3% risk of suicide attempt in the following month) and long-term (5-10 times greater risk for suicide and suicide attempt over the following 2 years) risks [11,12]. These risk levels are specific to PHQ item 9, which asks whether the patient, over the past 2 weeks, has been bothered by “thoughts that you would be better off dead or of hurting yourself in some way.” Responses of “more than half the days” or “nearly every day” are linked to the elevated risk described, and the risk remains to be significant when controlling for the total score of the remaining PHQ items and demographic variables. More recently, PHQ scores in combination with other EMR data have been used to predict suicide attempts and suicide using empirically derived risk scores [13].

A promising low-intensity intervention is caring messages, comprising brief, nondemanding, unsolicited caring messages sent by a health care provider over time. Although caring messages studies are not without limitations, data suggest that they may reduce the incidence of suicide in psychiatric patients [14,15]. It has also been shown to reduce additional suicide attempts [8,16-18]. Caring messages is among the few interventions that have replicated such findings [2,14,19-21]. The Institute of Medicine and the National Action Alliance for Suicide Prevention identify caring messages as a promising and recommended intervention [2,22], and it has been adapted for email [23]. A possible mechanism of action for caring messages is that it may address a lack of social connections [24].

DBT, another intervention, is a high-intensity treatment demonstrated to reduce suicidal behaviors by teaching individuals how to effectively manage intense emotions and tolerate distress [6]. Reductions in suicidal behaviors have been replicated across at least four DBT randomized controlled trials targeting suicidal individuals [19,25-28]. The key elements of DBT can be adapted for brief interventions. In a component analysis of DBT and a subsequent literature review, preliminary analyses suggest that one element—skills training—is effective in reducing suicidal behaviors [29,30]. Skills training has subsequently been adapted for brief, population-based interventions [31-34].

For the delivery of such interventions, the population-level impact requires a model that is affordable and scalable. Web-based interventions have the potential to reach large numbers of high-risk individuals and have been shown to be effective for support inside and outside medical settings [35-38]. Such interventions have demonstrated effects over usual care, in some cases comparable with in-person treatment [39-43]. Intervention websites, both with and without human support, are scalable for population-based uses in ways that in-person interventions are not [44]. Evidence supporting web-based adaptations of high-intensity treatments such as CBT warrants the development and testing of a web-based DBT skills intervention. The advantages of intervention websites include 24-hour accessibility, greater perceived privacy, and flexibility of location.

Meanwhile, EMR systems can be used not only to support the delivery of web-based interventions but also to identify those at risk and to support patient-provider secure messaging used for intervention. EMRs with secure messaging have the potential to significantly increase the efficiency and dissemination of suicide prevention and suicide attempts. Secure messaging has been successfully used to deliver low-intensity interventions [45-47]. Secure messaging can be used to support the delivery of web-based interventions traditionally delivered in person, such as CBT and motivational interviewing.

### Objectives

This pilot study aims to assess the feasibility, acceptability, and engagement of a brief suicide prevention intervention in preparation for a full-scale randomized trial. We evaluated this by measuring patient response to an invitation to a web-based, population-based suicide attempt and suicide prevention intervention, intended to be easily adapted into large health care systems, which included elements of DBT and caring messages.

## Methods

### Overview

We evaluated the feasibility, acceptability, and engagement of a brief web-based suicide prevention intervention using qualitative and quantitative methods. The intervention included EMR-mediated secure messaging linked to a web-based DBT skills platform. Participants included outpatients who were receiving care within Kaiser Permanente Washington, a health system serving approximately 700,000 people in Washington State, which routinely administers the PHQ to all patients receiving care for mental health conditions [10,48]. Patients were eligible if they were aged 18 years or older, had ID verification for secure email messaging through the EMR, had sent or read a secure message in the past year, and had completed a PHQ depression severity questionnaire in the previous week and responded “more than half the days” or “nearly every day” to item 9 (“Thoughts that you would be better off dead or of hurting yourself in some way”). Previous research in this setting indicates that this group would have an expected risk of suicide attempt of approximately 4% over the following year [11,12].

Over the course of 3.5 months, we used an automated EMR data query program to pull 6 samples, identify 60 individuals, and conduct outreach via secure messaging accessible on a mobile app or website. We used this sample size, which could be considered large for a pilot, to gain confidence in our estimates for the feasibility of a large clinical trial. We sent secure message invitations to the DBT skills program approximately every other week to between 5 and 20 eligible participants for each sample (starting at the top of the list) to evaluate ideal caseload management. Invitations were sent 1 to 2 weeks after the visit, if an elevated PHQ item 9 was involved. Patient care continued to be the responsibility of the behavioral health and primary care providers in the Kaiser Permanente Washington system who had administered the PHQ at the recent visit. In addition, a suicide risk protocol involving follow-up by study clinicians (author 1 or 3) for those who indicated imminent risk over secure message was in place, although the protocol was not triggered during the study. The study procedures were approved by the organization’s institutional review board, which included a waiver of research consent for patient identification and intervention invitation; however, this restricted the analysis of additional data (eg, descriptive data of eligible participants). The waiver of consent for patient identification and intervention invitation was granted because this pilot and subsequent trial involved no more than minimal risk and because the purpose of the pilot was to evaluate the feasibility and acceptability of procedures planned for the randomized trial of outreach. Evaluating only patients who actively consented to receive intervention would have yielded a result of questionable validity and generalizability.

The invitation messages, and the messages that followed, can be conceptualized as having two elements: a caring message (a brief and unsolicited expression of care) and support for a web-based DBT skills intervention (Textbox 1). We do not know whether the combination of these elements will be effective, which is not measured in this pilot. The footer of each message included the coach name and title and also included the option to opt out of additional messages, noting that messages were part of a research project and would be part of the EMR, and included numbers to call in case of immediate crisis. The secure message invitation included a link to a web-based video consent form (Multimedia Appendix 1). Patients who did not open the invitation received a telephone call. Those who opened it but did not visit the consent received a secure message reminder. Invitation messages were designed based on patient feedback that they would be most drawn to an intervention that made them feel cared for (eg, subject lines expressing concern), that was personalized, that others like them had found helpful, and that included examples with real people [49,50]. Once consent was confirmed, participants arrived at the landing page of the DBT skills site.

The web-based DBT skills site included four DBT skills (mindfulness, opposite action, mindfulness of current emotion, and paced breathing). This content focused on training in specific skills to manage upsetting or painful emotions that can precipitate suicidal thoughts and behavior. Content was based on a brief DBT skills face-to-face intervention developed and pilot-tested by the first author [33] and included a video-based series of didactic and personal story demonstrations of DBT skill use with a team of collaborators with lived experience of suicidal thoughts and behavior (Team Now Matters Now). DBT experts, including the DBT developer Marsha Linehan, also consulted on intervention development. The website landing page included a brief orientation video describing the intervention content and what to expect (Multimedia Appendix 2). Below the video on the landing page was a list of the four DBT skills, with a brief description (eg, “Mindfulness - A way to be present for what is most important in your life”; Textbox 2). Participants could also choose to learn more about Team Now Matters Now or provide feedback about the program. The DBT skills content was housed on a Health Insurance Portability and Accountability Act–compliant modular software platform (DatStat). Each of the four DBT skills followed the same path that included a 30-second overview or *teaser*, a 5- to 8-minute didactic video describing the skill as it might in a traditional DBT skills group, several 1- to 5-minute demonstration videos with real people talking about how they used the DBT skill in their own life, and a 1-page practice assignment similar to what one might find in a DBT skills group. One could return to the landing page to select another skill at any point in the pathway of a particular skill. In addition to the other benefits of web-based interventions outlined in the introduction, they also provide a consistent patient experience and are baked in fidelity [51].

**Secure message invitation.**

Dear [First Name]

Sometimes a little extra help can be just what someone needs to get through tough times.

A new web-based program called Now Matters Now was designed to give you that help when you need it. The program uses real people to teach specific coping skills, like mindfulness and paced breathing.

We invite you to try Now Matters Now today. Learn more at <Link to Intervention Website>

We care about you.

My job is to encourage you to use the program over the next three months and to practice the skills you are learning. If you haven’t visited the program in a while, I’ll send you a message to remind you. If you have visited, I’ll check with you about what you find helpful.

I’ll communicate with you through messages, and in order to message you, I will access your medical record.

Sending care,

**Dialectical behavior therapy skills site landing page text.**

- TEAM NOW MATTERS NOW—Introducing the real people who use these skills and helped develop this program
- MINDFULNESS—A way to be present for what is most important in your life
- OPPOSITE ACTION—Living a full and healthy life, despite what negative or unhelpful emotions and thoughts are telling you to do
- MINDFULNESS OF CURRENT EMOTION—Observing, honoring, and moving through strong emotions without being controlled by them
- PACED BREATHING—A different and scientific approach to the old saying “Just Breathe”
- GIVE US FEEDBACK—Did you have technical problems with this program? Do you want to give us feedback about the program, good or bad?

The DBT skills intervention was supported by an interventionist or program *coach* for 3 months. In line with the stage model of behavior therapies, the interventionist for this study was the lead intervention developer (UW) [51-53]. This included caring messages outreach and support for the use of the DBT skills site. For 2 months, the coach delivered this support exclusively via secure messaging, reaching out approximately once per week for participants’ engagement and less frequently for those who had not responded to the most recent secure message. For the third month, the patient could continue to engage in the intervention or send the coach messages and the coach would reply, but the coach would not actively outreach or prompt the patient. The intervention coach did not provide psychotherapy but instead provided caring messages as well as outreach in the form of reinforcement and contingency management surrounding the use of the DBT skills site. This would include following up with patients who had not visited the site in several weeks, suggesting specific skills they might find useful or thanking the patient for visiting the site and include a tip or point relevant to the DBT skill they viewed (Textbox 3 provides an example of this type of message). The research team met weekly with the senior investigator (author GES) to ensure safety monitoring and coach fidelity. The senior investigator also had access to review the secure messages of the coach to monitor secure message fidelity to the described approach.

**Coach outreach/and support message example.**

Hi [First Name],

I’m writing to check-in from the Now Matters Now program. Do any of the skills look interesting to you to try?

Paced Breathing is a breathing technique that can calm the mind and body naturally. Some people like to use Paced Breathing when they’re feeling nervous or anxious. It can also be a helpful way to clear the mind if you’re having a difficult time falling asleep.

The key to Paced Breathing is to have your exhale be longer than your inhale. It can be a good way to center yourself and makes it easier to then practice other skills.

If you try it, let me know how it goes.

To learn more, please click the link below:

[link to Consent “form” video]

Take care,

### Feasibility, Acceptability, and Engagement

We evaluated feasibility by measuring the proportion of invited patients who opened the program invitation, the proportion of patients who visited the consent page, and the proportion of patients who consented. We qualitatively evaluated acceptability by using secure message feedback that the coach received from patients invited to participate and summarized relevant themes [54]. We evaluated engagement by measuring the proportion of participants who began using the DBT skills site and the number of site visits. We also measured visits to each of the featured DBT skills in the program among a subset of the sample, and the first 20 participants were invited. We did not collect engagement data beyond the first 20 patients eligible for this pilot study because the original purpose of these analyses was to help confirm that the training website was working as planned (to help evaluate the feasibility of a large randomized trial).

## Results

### Feasibility

**Figure 1:**
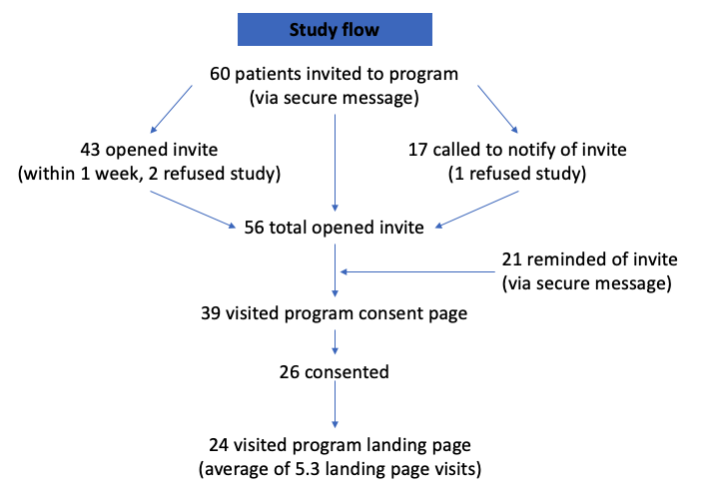
Study flow.

In total, 60 patients were invited to participate (Figure 1). Of these 60 patients, 17 (28%) patients did not open the invitation within 1 week of delivery and received a telephone call (10 were left a voicemail and 7 were spoken to). Up to 2 secure message reminders were sent to 35% (21/60) of patients. Overall, 3% (2/60) of patients refused to participate. Furthermore, 93% (56/60) opened the invitation (indicating that these people did, as far as we know, read and *receive* the caring message aspect of this contact), 65% (39/60) clicked the link to the consent form, and 43% (26/60) consented to participate.

### Acceptability

The coach received no complaints from those invited to participate in the program. The feedback the coach received via secure messaging mainly included descriptions of how participants had used the program, specifically their helpful experiences practicing each of the four program skills and the challenges they experienced. One participant described a helpful experience in trying mindfulness skills, which involved bringing attention and awareness to their thoughts:

I did the first lesson last night—I was able to use it today when I started having negative thoughts that wouldn’t stop. I cleared my mind and started looking at the trees, buildings and other things around me and noticing the little details of things. It actually calmed me down and gave me a sense of control.

Another participant described the experience of practicing paced breathing:

I am doing my best to make the Paced Breathing a daily practice. I forget sometimes until I am “over the edge” and am in deep panic/terror, but I can still even then use the breathing to help get me feeling more calm.

Another participant described a powerful experience using an opposite action, which involved choosing to do the opposite of ineffective emotional urges:

Yesterday I began the module called “Opposite Action” and printed it out after I completed it. Doing it was empowering but also very emotional for me.

Finally, a participant described a plan to use mindfulness of current emotion, which involved learning how to bring attention and awareness to the bodily sensations of emotions:

I used [a DBT skill] about 5 times today when I was out walking—it keeps my emotions under control, so I don’t start crying. I’m going to try [the]“mindful emotion” thing tomorrow. I appreciate [my health care organization] offering something like this—I like having concrete tools I can use.

In terms of challenges, participants described difficulties in using their skills effectively while experiencing intense emotions. For example, one participant described:

One inescapable problem I have found is that once the line that gets crossed where “there is no hope” of being able to turn around the anxiety, I have little ability to do anything constructive to stop the terror from escalating ... if I am not too “far gone” the skills do help to lower the physical and mental/emotional distress.

### Engagement

In total, 92% (24/26) of the participants that consented had visited the landing page of DBT skills. These DBT skills site users logged in on an average of 5.3 times to the home page (SD 6.0, range 1-26). We examined a subsample of those consenters to look at engagement in the specific DBT modules (eg, opposite action). Among the first 20 participants enrolled, 19 (95%) read the invite. Of these 19 participants, 12 (63%) visited the website and 8 (42%) agreed to participate. Of those who consented, participant engagement in the different skills ranged from 38% (3/8) for paced breathing to 88% (7/8) for opposite action (Table 1).

**Table 1:** Dialectical behavior therapy skill engagement for subsample (N=8).

Program visits	Opposite action	Mindfulness	Mindfulness of current emotion	Paced breathing
Visited at least once, n (%)	7 (88)	4 (50)	6 (75)	3 (38)
Average visit, range (min)	23 (1-54)	34 (2-65)	13 (1-57)	18 (3-30)
Visited at least twice, n (%)	4 (50)	3 (38)	1 (13)	3 (38)
Visited 3 times or more, n (%)	2 (25)	3 (38)	1 (13)	1 (13)

## Discussion

### Principal Findings

This pilot study demonstrated the feasibility, acceptability, and engagement of a population-based intervention involving active elements of caring messages and DBT. Specifically, we demonstrated that by using an accurate method for identifying those at risk for suicide and suicide attempt—in this case, PHQ item 9—we can adapt existing interventions to be suitable for delivery on a large scale and that these models are promising for intervention delivery.

The number of patients who received the initial message that involved elements of caring messages was high (56/60, 93%). This provides support for the delivery of caring messages through this type of intervention (delivered over time through secure messaging with the support of a coach or care manager). The rate of patients who actually reached the DBT skills site content (26/60, 43%) indicates a need for strategies to reduce barriers to DBT skills site participation, such as simplifying the invitation and consent process for patients. The steepest drops were between viewing the message and visiting the consent page (17/56, 30% of those remaining were lost), and between visiting the consent page and consenting to the DBT skills intervention (13/39, 33% of those remaining were lost; Figure 1). Although it is reasonable to hypothesize that those dropping off were concerned about loss of autonomy or stigma [55,56], participants eligible for this study had already made a significant suicidal disclosure in endorsing suicidal thoughts at a recent visit. Furthermore, while the consent video explicitly refers to suicidal thoughts, the invitation message did not. Indeed, patients did not know that was part of the reason they were approached until they reached the consent video. Notably, all patients in the program had already received care in the health system. It is certainly possible that some patients declined to participate because they were already satisfied with their care. Patients who gave consent to intervention participation appeared to engage, and some gave positive feedback about the content of the intervention.

### Limitations

A limitation of our study was that the data analysis was limited to anonymized information regarding program participation. Health records data to identify potential participants were used under a waiver of informed consent, so we did not retain demographic, diagnostic, or other clinical information, including the total PHQ score. Although patients were identified as eligible based on their high severity of suicidal thoughts and all eligible patients were enrolled in the study, it could be that there were more and less severe patients within this group and that DBT skills intervention participation varied depending on the severity. Furthermore, participation among those invited may have been reduced by the need for a research consent process. We suspect that engagement would have been higher without this step between secure message invitation and the DBT skills site. In addition, we do not yet know whether the combination of elements of caring messages with brief DBT skills support will be effective, given that caring messages are intended to be nondirective. Finally, it is possible that those who received the invitation to the intervention felt singled out in some way, having had a visit recently where they disclosed suicidal thoughts. Indeed, qualitative research indicates that those who receive the PHQ-9 have significant concerns about how the information regarding the ninth item and suicide will be used [55,56]. Instead, the invitation might read, “As part of my job I reach out to many people we think might benefit from this program....” The key would be to not add too much text for patients to read, while remaining mindful of potential patient fears associated with suicide or mental health–related care (eg, loss of autonomy and psychiatric hospitalizations) [56,57]. Furthermore, our sample of engagement data was small (n=20). It would have been helpful to collect engagement data for all patients invited to participate in the intervention.

As noted by one participant, once one’s emotions reach a certain threshold, the DBT skills become more difficult to access. One limitation of this intervention is that the DBT skills content was not framed in terms of skills for day-to-day stressors versus skills for acute crises. Future interventions may benefit from tailoring additional skills to match the spectrum of emotional stress/crises [58].

### Conclusions

This project addresses a number of the most important conceptual, methodological, logistical, and ethical challenges that hamper major advancement in the field of suicide prevention. Qualitative and quantitative data from this study were used to inform the design of a full-scale effectiveness trial (ClinicalTrials.gov: NCT02326883). This research, led by four health care systems within the Mental Health Research Network, is underway to determine its effectiveness [59]. In that trial, participants are randomly assigned to continued usual care, an outreach-based care management intervention, or a coach-supported, web-based DBT skills training program with elements of caring messages, based on the intervention developed and evaluated here.

The videos that were developed as part of this project that make up most of the DBT skills content are publicly available to both the researchers and the public as one piece of NowMattersNow.org [60]. Survey participants to this website have described reductions in suicidal thoughts and negative emotions while visiting the website [34]. Although brief web-based interventions cannot replace intensive tertiary interventions (eg, in-person DBT/CBT), they hold promise as a low-intensity, population-based intervention by ensuring that the right content is consistently delivered to individuals at high risk of suicide and suicide attempt at the time it is needed.

## Appendix

Multimedia Appendix 1 Consent “form” video.

Multimedia Appendix 2 Program landing page video.

## Abbreviations

CBT: cognitive behavioral therapy
DBT: dialectical behavior therapy
EMR: electronic medical record
PHQ: Patient Health Questionnaire
